# Supplementary material for: Discovery and pharmacophoric characterization of chemokine network inhibitors using phage-display, saturation mutagenesis and computational modelling
Source: Nat Commun. 2023 Sep 16;14:5763. doi: 10.1038/s41467-023-41488-z (PMC10505172; doi:10.1038/s41467-023-41488-z)
Supplement: Supplementary file 4 — Source Data [file 41467_2023_41488_MOESM4_ESM.zip › SourceData/scripts/xoctet_20230808081637.tar!/xoctet/analysis/kinetic/SV_E19_sHD2_CCL11_kinetic_plot.pdf]

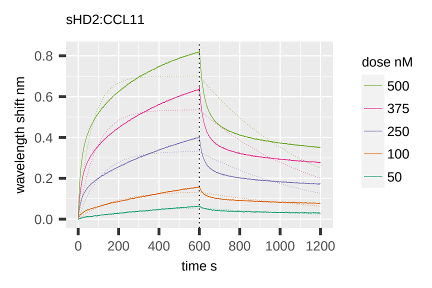

sHD2:CCL11
wavelength shift nm
0.0
0.2
0.4
0.6
0.8
0
200
400
600
800
1000
1200
time s
dose nM
500
375
250
100
50
